# Alzheimer’s disease genetic risk and changes in brain atrophy and white matter hyperintensities in cognitively unimpaired adults

**DOI:** 10.1093/braincomms/fcae276

**Published:** 2024-08-14

**Authors:** Anja Soldan, Jiangxia Wang, Corinne Pettigrew, Christos Davatzikos, Guray Erus, Timothy J Hohman, Logan Dumitrescu, Murat Bilgel, Susan M Resnick, Leonardo A Rivera-Rivera, Rebecca Langhough, Sterling C Johnson, Tammie Benzinger, John C Morris, Simon M Laws, Jurgen Fripp, Colin L Masters, Marilyn S Albert

**Affiliations:** Department of Neurology, Johns Hopkins University School of Medicine, Baltimore, MD 21205, USA; Department of Biostatistics, Johns Hopkins Bloomberg School of Public Health, Baltimore, MD 21205, USA; Department of Neurology, Johns Hopkins University School of Medicine, Baltimore, MD 21205, USA; Centre for Biomedical Image Computing and Analytics, Perelman School of Medicine, University of Pennsylvania, Philadelphia, PA 19104, USA; Centre for Biomedical Image Computing and Analytics, Perelman School of Medicine, University of Pennsylvania, Philadelphia, PA 19104, USA; Department of Neurology, Vanderbilt University Medical Center, Nashville, TN 37212, USA; Department of Neurology, Vanderbilt University Medical Center, Nashville, TN 37212, USA; Laboratory of Behavioral Neuroscience, National Institute on Aging Intramural Research Program, Baltimore, MD 21224, USA; Laboratory of Behavioral Neuroscience, National Institute on Aging Intramural Research Program, Baltimore, MD 21224, USA; Wisconsin Alzheimer's Disease Research Center, University of Wisconsin-Madison School of Medicine and Public Health, Madison, WI 53726, USA; Wisconsin Alzheimer's Disease Research Center, University of Wisconsin-Madison School of Medicine and Public Health, Madison, WI 53726, USA; Wisconsin Alzheimer's Disease Research Center, University of Wisconsin-Madison School of Medicine and Public Health, Madison, WI 53726, USA; Knight Alzheimer Disease Research Center, Washington University School of Medicine, St. Louis, MO 63110, USA; Knight Alzheimer Disease Research Center, Washington University School of Medicine, St. Louis, MO 63110, USA; Centre for Precision Health, Edith Cowan University, Joondalup, WA 6027, Australia; Australian E-Health Research Centre, CSIRO Health & Biosecurity, Herston, QLD 4029, Australia; The Florey Institute, University of Melbourne, Parkville, VIC 3052, Australia; Department of Neurology, Johns Hopkins University School of Medicine, Baltimore, MD 21205, USA

**Keywords:** Alzheimer’s disease (AD), *APOE*, polygenic risk score (PRS), magnetic resonance imaging (MRI), white matter hyperintensities

## Abstract

Reduced brain volumes and more prominent white matter hyperintensities on MRI scans are commonly observed among older adults without cognitive impairment. However, it remains unclear whether rates of change in these measures among cognitively normal adults differ as a function of genetic risk for late-onset Alzheimer’s disease, including *APOE*-ɛ4, *APOE*-ɛ2 and Alzheimer’s disease polygenic risk scores (AD-PRS), and whether these relationships are influenced by other variables. This longitudinal study examined the trajectories of regional brain volumes and white matter hyperintensities in relationship to *APOE* genotypes (*N* = 1541) and AD-PRS (*N* = 1093) in a harmonized dataset of middle-aged and older individuals with normal cognition at baseline (mean baseline age = 66 years, SD = 9.6) and an average of 5.3 years of MRI follow-up (max = 24 years). Atrophy on volumetric MRI scans was quantified in three ways: (i) a composite score of regions vulnerable to Alzheimer’s disease (SPARE-AD); (ii) hippocampal volume; and (iii) a composite score of regions indexing advanced non-Alzheimer’s disease-related brain aging (SPARE-BA). Global white matter hyperintensity volumes were derived from fluid attenuated inversion recovery (FLAIR) MRI. Using linear mixed effects models, there was an *APOE*-ɛ4 gene-dose effect on atrophy in the SPARE-AD composite and hippocampus, with greatest atrophy among ɛ4/ɛ4 carriers, followed by ɛ4 heterozygouts, and lowest among ɛ3 homozygouts and ɛ2/ɛ2 and ɛ2/ɛ3 carriers, who did not differ from one another. The negative associations of *APOE*-*ɛ*4 with atrophy were reduced among those with higher education (*P* < 0.04) and younger baseline ages (*P* < 0.03). Higher AD-PRS were also associated with greater atrophy in SPARE-AD (*P* = 0.035) and the hippocampus (*P* = 0.014), independent of *APOE*-ɛ4 status. *APOE*-ɛ2 status (ɛ2/ɛ2 and ɛ2/ɛ3 combined) was not related to baseline levels or atrophy in SPARE-AD, SPARE-BA or the hippocampus, but was related to greater increases in white matter hyperintensities (*P* = 0.014). Additionally, there was an *APOE*-ɛ4 × AD-PRS interaction in relation to white matter hyperintensities (*P* = 0.038), with greater increases in white matter hyperintensities among *APOE*-ɛ4 carriers with higher AD-PRS. *APOE* and AD-PRS associations with MRI measures did not differ by sex. These results suggest that *APOE*-*ɛ*4 and AD-PRS independently and additively influence longitudinal declines in brain volumes sensitive to Alzheimer’s disease and synergistically increase white matter hyperintensity accumulation among cognitively normal individuals. Conversely, *APOE*-*ɛ*2 primarily influences white matter hyperintensity accumulation, not brain atrophy. Results are consistent with the view that genetic factors for Alzheimer’s disease influence atrophy in a regionally specific manner, likely reflecting preclinical neurodegeneration, and that Alzheimer’s disease risk genes contribute to white matter hyperintensity formation.

## Teaser

Soldan *et al*. examined Alzheimer’s disease genetic risk in relation to changes in brain volumes and white matter lesions in cognitively unimpaired adults. The *APOE*-*ɛ*4 gene and Alzheimer’s disease polygenic risk scores independently influenced atrophy and synergistically influenced white matter lesions. The *APOE*-*ɛ*2 gene increased white matter lesions, not atrophy.

## Graphical Abstract

**Graphical Abstract:**
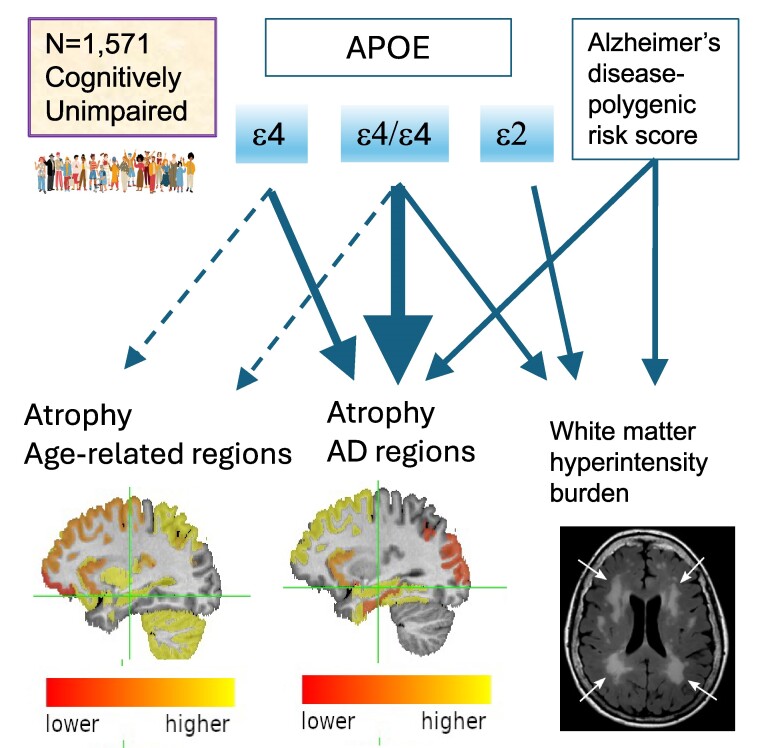

## Introduction

Alzheimer’s disease (AD) pathology and neurodegeneration, as measured by atrophy on magnetic resonance imaging (MRI), are present many years prior to the emergence of clinical symptoms when individuals are cognitively normal.^1,2^ Older adults without cognitive impairment also frequently have evidence of small vessel cerebrovascular disease, which most commonly manifests as white matter hyperintensities (WMHs) on MRI scans.^3^ Recent evidence suggests that WMH may also play a role in Alzheimer's disease,^4,5^ with both vascular and Alzheimer’s disease-specific pathways contributing to WMHs.^6,7^ Both brain atrophy^8,9^ and WMH burden^10,11^ among individuals with normal cognition have been shown to predict subsequent cognitive decline and impairment. It remains unclear, however, whether rates of brain atrophy and WMH accumulation among cognitively normal adults differ as a function of genetic risk for late-onset Alzheimer’s disease and whether this relationship is influenced by other variables, such as age, sex, vascular risk factors and education. This is an important topic for investigation because an examination of non-modifiable and modifiable factors that influence longitudinal changes in atrophy and WMH may help identify ways to reduce brain deterioration and eventual cognitive decline in older persons.

The major genetic risk factor for late-onset Alzheimer’s disease is the apolipoprotein E (*APOE*) gene, with the ɛ4 allele increasing risk of dementia^12,13^ and the ɛ2 allele decreasing risk.^14,15^ Multiple additional genetic loci have been identified in genome-wide association studies (GWAS)^16^ to increase late-onset Alzheimer’s disease-dementia risk, though to a smaller degree than *APOE*-ɛ4. To assess the cumulative impact of these other genetic loci on dementia risk, they are often combined into polygenic risk scores for Alzheimer’s disease (AD-PRS).^17-20^

Many prior studies investigating Alzheimer’s disease-genetic risk in relation to brain atrophy or WMH accumulation have included a mixture of participants across the clinical spectrum [i.e. cognitively normal, mild cognitive impairment (MCI) and dementia] or non-demented cohorts (i.e. cognitively normal and MCI). Taken together, these studies suggest that *APOE*-ɛ4 genetic status^21-27^ and higher AD-PRS scores^21,24,26,28,29^ are both associated with lower volumes or thickness of Alzheimer’s disease-vulnerable regions, with higher rates of atrophy in these regions,^18,30-36^ and with higher levels of^37-41^ and greater increases in WMH burden over time^27,42,43^ (but see Tank *et al*.,^24^ Habes *et al*.,^44^ Lyall *et al*.,^45^ Lane *et al*.^46^ and Debette *et al*.^47^).

Research among middle-aged and older individuals with normal cognition, however, has primarily included cross-sectional studies that cannot address whether observed differences in brain volumes as a function of Alzheimer’s disease-genetic risk reflect lifelong differences in brain structure, as opposed to differential atrophy that occurs during the preclinical phase of Alzheimer’s disease. Results from cross-sectional studies have been mixed, with some finding higher WMH burden^48,49^ and lower volumes or thinner cortex^23,50-53^ among individuals at greater Alzheimer’s disease-genetic risk (i.e. *APOE*-ɛ4 carriers and/or higher AD-PRS), and others not finding such differences.^23,54-59^ For *APOE*-ɛ2 genetic status, results from cross-sectional studies have also been mixed.^22,52,54,60,61^ Few prior longitudinal studies have been conducted among cognitively unimpaired older individuals. Of these, two reported greater volume loss in Alzheimer’s disease-vulnerable regions among *APOE*-ɛ4 carriers compared to non-carriers,^62,63^ whereas two others found no *APOE*-ɛ4-related differences.^64,65^ Additionally, a relatively small study reported less hippocampal atrophy among older cognitively normal *APOE*-ɛ2 carriers relative to ɛ3 homozygouts,^66^ consistent with a study that included individuals across the Alzheimer’s disease-spectrum.^33^ To our knowledge, the relationship of AD-PRS and longitudinal atrophy rates or WMH accumulation have not been examined among middle-aged and older cognitively unimpaired individuals. Likewise, although cross-sectional studies across the AD-spectrum have found higher WMH burden in *APOE*-ɛ2 carriers relative to ɛ3/ɛ3 homozygotes,^40^ the impact of the *APOE*-ɛ2 allele on longitudinal changes in WMH burden in cognitively normal individuals remains unclear.

To address these gaps, the current study examined rates of change in regional brain volumes and WMH in a large, harmonized dataset of middle-aged and older individuals with normal cognition at baseline (mean MRI follow-up = 5.3 years, max = 24 years), with both *APOE* genotypes (*N* = 1541) and AD-PRS scores (*N* = 1093) available. The current study expands on prior ones in several ways. First, prior studies have been characterized by either short follow-up periods (i.e. mean follow-up 2–3.5 years),^63-66^ or by small sample sizes (i.e. *N* < 110),^62,65,66^ limiting their ability to draw inferences regarding less frequent alleles, including ɛ2 carrier status and ɛ4 homozygosity. Second, the large sample size allowed us to examine potential interactions between Alzheimer’s disease-genetic risk and other variables in relationship to brain atrophy or WMH accumulation, including age, sex, education, vascular risk and progressor status (i.e. remained cognitively normal versus progressed to MCI or dementia). Third, we examined atrophy rates in three different measures: (i) a composite score of Alzheimer’s disease-vulnerable regions derived from machine learning (SPARE-AD); (ii) hippocampal volume; and (iii) a composite score of regions sensitive to non-Alzheimer’s disease-related aging (SPARE-BA), also derived from machine learning. This allows for a comparison of the influence of Alzheimer’s disease-genetic risk on atrophy in Alzheimer’s disease-vulnerable and non-vulnerable regions. Lastly, we examined the impact of AD-PRS on longitudinal brain atrophy and WMH change, as well as interactions between AD-PRS and *APOE* genotypes on these measures.

## Materials and methods

### Participants

This study used data from the Preclinical Alzheimer’s disease Consortium (PAC), a multi-site collaboration established to investigate the earliest phases of Alzheimer’s disease. The PAC study includes harmonized cognitive, clinical, genetic, MRI and amyloid imaging data from five on-going longitudinal cohort studies: the Adult Children Study (ACS),^67^ the Australian Imaging, Biomarker, and Lifestyle study (AIBL study),^68^ the Biomarkers of Cognitive Decline Among Normal Individuals (BIOCARD) study,^69^ the Neuroimaging Substudy of the Baltimore Longitudinal Study of Aging (BLSA)^70^ and the Wisconsin Registry for Alzheimer’s Prevention (WRAP).^71^ To be included in the PAC data files, each participant had to be cognitively normal at baseline and have at least one molecular biomarker (derived from cerebrospinal fluid or positron emission tomography) collected while they were cognitively normal. By design, at least half of the participants in each cohort, except BLSA, had a family history of dementia. Individuals with epilepsy, recent strokes or remote strokes with residual effects were excluded at baseline. Additional details regarding study design and inclusion/exclusion criteria have been published previously for each cohort.^67,69-72^ Molecular biomarkers were not considered in the present analyses. Participants in all cohorts provided written informed consent according to the Declaration of Helsinki. The study protocols were approved by each site’s local institutional review board.

### Clinical and cognitive assessments

Participants in all cohorts undergo longitudinal clinical and cognitive assessments, as well as medical, neurologic and psychiatric evaluations at regular intervals, depending on the protocol for each site (e.g. every 12, 18 or 24 months). The cognitive assessments at each site include a comprehensive neuropsychological battery covering all major cognitive domains (for details, see Gross *et al*.^73^ and Pettigrew *et al*.^74^). All sites conduct regular consensus diagnoses for all participants using published criteria, e.g. the National Institute on Aging/Alzheimer’s Association criteria for MCI^75^ and dementia.^76^

The diagnostic process for each case is handled in a comparable manner at each site: (i) clinical data are examined pertaining to the medical, neurologic and psychiatric status of the subject; (ii) reports of changes in cognition by the subject and by collateral sources are examined, based on the Clinical Dementia Rating scale; and (iii) change in cognitive performance is established. This information is then used to determine whether the subject has become cognitively impaired, and determine the likely aetiology of the impairment. Clinical diagnoses were made without knowledge of the biomarker measures. To be included in the current analyses, participants had to be cognitively normal at the time of their first MRI scan (which is considered the ‘baseline’ in these analyses) and have non-missing *APOE* genetic and vascular risk score data (see below). Participants with a diagnosis of ‘impaired not MCI’ were included with the cognitively normal participants, as they do not meet criteria for MCI, consistent with prior publications.^69^

Summary vascular risk scores were calculated using a previously validated method, based on the presence or absence of five vascular risk factors: hypertension, diabetes, obesity (defined as a body mass index > 30 kg/m^2^), hypercholesterolaemia and smoking within the 30 days prior to data collection.^77^ This information was obtained from medical history reports or medical records collected at visits coinciding with the MRI visits (±12 months). The risk factors were coded dichotomously (0 if absent and 1 if present or remote) and then summed to calculate summary scores (max = 5) for each visit, consistent with prior publications.^78-80^

### Genetic measures

Participants at each site provided blood that was used for DNA extraction. *APOE* alleles were determined using standard targeted genotyping, i.e. by direct genotyping (rs7412 and rs429358) in ACS, WRAP and AIBL, or by restriction isotyping (codon 112 and 158) in BIOCARD and BLSA. Dichotomous indicators were created for *APOE*-ɛ2 carriers (ɛ2/ɛ2 and ɛ2/3 = 1; otherwise 0), *APOE*-ɛ3/ɛ3 homozygous carriers (ɛ3/ɛ3 = 1, otherwise 0) and *APOE*-ɛ4 carriers (ɛ2/ɛ4, ɛ3/ɛ4 and ɛ4/ɛ4 = 1; otherwise 0). Participants with ɛ2/ɛ4 alleles were included in the *APOE*-ɛ4 group given their risk for AD pathology is similar to that of ɛ4 carriers, rather than ɛ2 carriers.^81^ An additional categorical variable for *APOE*-ɛ4 carrier status was also created to examine potential differences between ɛ4 homozygous versus heterozygous individuals (i.e. ɛ2/ɛ2 and ɛ2/ɛ3 versus ɛ3/ɛ3 versus ɛ3/ɛ4 versus ɛ4/ɛ4). The ɛ2/ɛ2 was combined with the ɛ2/ɛ3 group due to their sample size (*n* = 7 across all sites).

Details regarding the generation of the AD-PRS for the PAC dataset have been described previously.^74^ Briefly, each site generated GWAS data using various genotyping arrays and the raw GWAS data were imputed by chip using a standard pipeline that included variant filtering for genotyping efficiency (95%), minor allele frequency (>1%) and Hardy-Weinberg equilibrium (*P* > 1 × 10^−6^). Given the racial and ethnic makeup of the included studies, all GWAS analyses were restricted to those of European ancestry that was confirmed using population principal component analysis. For the purpose of the AD-PRS analysis, we restricted all GWAS datasets to overlapping variants leaving a total of 6 739 456 common variants available in all five datasets for analysis. AD-PRS were generated using imputed GWAS data, leveraging the summary statistics provided by Kunkle *et al*.^16^ that were regenerated for us removing PAC participants who were included in the original GWAS analysis (*n* = 93 220). AD-PRS were computed with PLINK using a previously published method.^18^ The current analyses only used AD-PRS without the *APOE* region (i.e. 1 MB upstream and downstream of the *APOE* gene) to assess the independent associations of *APOE* and other Alzheimer’s disease risk genes on the MRI measures. AD-PRS were transformed to *Z*-scores to simplify interpretation, using the mean and standard deviation (SD) across all five datasets (see Pettigrew *et al*.^74^ for the distribution of harmonized AD-PRS in the PAC cohorts).

### MRI assessments

#### Image acquisition

All PAC sites have collected structural MRI scans longitudinally, with the majority of scans acquired on 3 T scanners, but a subset on a 1.5 T scanner, since some of the studies began in the mid-1990s. See Supplementary Table 1 for details regarding the types of scanners and acquisition protocols for each site.

#### Image processing and harmonization

Processing of T_1_-weighted images included correction of intensity inhomogeneities,^82^ skull stripping^83^ and segmentation of the brain into a set of anatomical regions of interest (ROIs) using the Multi-atlas Region Segmentation Utilizing Ensembles (MUSE) software platform.^84^ This method was specifically designed for longitudinal studies to handle differences in scanners and imaging protocols over time and across sites and employs harmonized acquisition-specific atlases. For a detailed description of these methods, see Erus *et al*.^85^ and Habes *et al*.^86^ Briefly, MUSE uses a consensus labelling framework that combines an ensemble of labelled atlases in target image space by using multiple atlases reflecting a broad representation of anatomy. Scanner-specific atlases share the same ROI labels, imposing consistency of segmentations, while each atlas set preserves the image intensity characteristics of the specific scanner. The MUSE pipeline has been extensively validated against benchmark methods and applied in various cross-sectional and longitudinal studies.^64,84,86,87^ In comparison to most commonly used segmentation tools, such as FreeSurfer, MUSE has demonstrated significant improvement in accuracy and more consistent segmentations across scanners, particularly in segmentation of deep brain structures.^88^ The MUSE software package is freely available: https://www.med.upenn.edu/cbica/sbia/muse.html.

Additional statistical harmonization was applied to the ROI volumes based on the multivariate ComBAT-GAM method^89^ to remove cohort-related effects and protocol-specific variability. This method simultaneously models scanner effects (unwanted sources of variation) and covariate associations (e.g. age and sex). This harmonization approach integrates a generalized additive model, with a smoothed non-linear term for age, using thin plate regression splines, and linear terms for sex and intra-cranial volume (ICV), thereby preserving age and sex differences across sites.^89^

Quantification of WMH volumes from fluid attenuated inversion recovery (FLAIR) images was completed using an automated deep learning based segmentation method^90^ that is built upon the UNet architecture,^91^ with the convolutional network layers replaced by an Inception ResNet architecture.^92^ The network model uses inhomogeneity corrected and co-registered FLAIR and T_1_-weighted images as input, and has been trained using a multi-site training dataset with human-validated WMH labels, as published previously.^86^ The algorithm was applied to MRI scans of PAC participants to calculate binary WMH masks, and to extract regional WMH volumes. The current analyses used global WMH volumes.

#### Volumetric regions of interest and spatial patterns of atrophy

Harmonized volumes of the left and right hippocampus were normalized for head size by regressing the average of the left and right hemispheres on ICV. The standardized residuals (mean = 0, SD = 1) were used in analyses presented below. Hippocampal volumes were examined to enable direct comparison to many prior studies that have specifically focused on this structure.

Atrophy in regions vulnerable to Alzheimer's disease was measured using SPARE-AD scores, which represent an imaging signature of Alzheimer's disease-like neurodegeneration derived from machine learning, as previously described and validated.^28,93^ For SPARE-AD calculation, a support vector machine classifier with a linear kernel was trained to maximally differentiate between cognitively unimpaired participants and participants with AD-dementia, using a curated dataset of over 10 000 individuals, known as the iSTAGING consortium,^86^ which includes the PAC sites. More positive SPARE-AD scores imply a more Alzheimer's disease-like brain structure (i.e. more AD-related atrophy).

We also calculated a brain signature of age-related brain atrophy, using SPARE-BA scores, to estimate structural brain changes due to aging. As published previously, this MRI approach uses a multivariate pattern regression method based on support vector regression to calculate brain aging scores for each participant.^86,94^ The model was trained with the T_1_-MR scans using harmonized ROI volumes for structures. In the present analyses, SPARE-BA scores were regressed on age at scan and the standardized residuals (referred to in the tables as SPARE-BA-resid) were used in the analyses presented below, with more positive scores indicating greater age-related atrophy compared to normative trends. This is comparable to ‘brain age gap’ scores estimated using related techiques.^95,96^ The regions contributing to the SPARE-BA scores are weighted optimally for estimating age, whereas the regions contributing to SPARE-AD scores are weighted optimally to distinguish between cognitively normal individuals and individuals with Alzheimer's disease-dementia. See Supplementary Fig. 1 for illustrations of the SPARE-AD and SPARE-BA masks and Supplementary Text 1 for additional information on SPARE-AD and SPARE-BA.

### Statistical analyses

We used linear mixed effects models with random intercepts and slopes with an unstructured covariance to evaluate whether the trajectories of the MRI measures of atrophy (i.e. SPARE-AD and SPARE-BA), hippocampal volume or WMH burden differed based on AD-genetic risk. Separate models were run for each MRI outcome measure. The primary models evaluating *APOE* effects included the following predictors: baseline age, sex, education, dichotomous indicators for *APOE*-ɛ2 and *APOE*-ɛ4 (with ɛ3/ɛ3 as the reference group), indicators for site (to control for potential site differences), time and the interaction (cross-product) of each predictor with time. The primary models evaluating AD-PRS were the same as the *APOE* models, but additionally included the AD-PRS. The years of education variable was standardized to *Z*-scores separately for cohorts within versus outside the USA, given differences in the number of years of compulsory schooling. Final models examining the trajectories of the atrophy measures and hippocampal volume included a time^2^ term to account for their statistically significant non-linear change over time. For the WMH models, the time^2^ was not significant and therefore not included. The primary *APOE* models were re-run with *APOE*-ɛ4 status coded categorically (as described above) to evaluate whether the MRI trajectories differed between *APOE* ɛ3/ɛ4 and ɛ4/ɛ4 carriers. In a sensitivity analysis, the primary models were also re-run excluding *APOE* ɛ2/ɛ4 carriers.

A second set of linear mixed effects models evaluated whether the results remained the same when additionally covarying both vascular risk summary scores (using all available measures over time) and participants’ follow-up diagnostic status, based on their last (i.e. most recent) consensus diagnosis (coded as 0 = remained normal, or 1 = progressed to MCI or dementia). These models were identical to the primary models, but additionally included the vascular risk summary scores over time and binary indicators for progressor status, as well as their interactions with time.

Lastly, to evaluate whether the relationships between the Alzheimer's disease-genetic factors and the MRI measures were modified by the demographic or clinical variables, a third set of linear mixed effects models were run. These models were the same as the primary models, but additionally included three-way interaction terms for the genetic factors × demographic/clinical variable × time (e.g. *APOE*-ɛ2 × baseline age × time and ɛ4 × baseline age × time; or AD-PRS × baseline age × time), as well as the corresponding lower-order interaction terms. These models were not adjusted for multiple comparisons because they were exploratory in nature and correcting for multiple comparisons in exploratory analyses can increase the likelihood of Type II errors and potentially obscure meaningful findings.

Data analysis was performed using STATA 17.0 and *P*-values of <0.05 were considered significant.

## Results

Table 1 shows baseline characteristics of participants included in the MRI volumetric and WMH analyses, separately for participants in the *APOE* and AD-PRS analyses. For baseline characteristics by cohort, see Supplementary Tables 2 and 3. On average, participants were in their mid-60s at baseline, primarily White and highly educated. About one-third of participants were *APOE*-ɛ4 carriers and approximately two-thirds had one or more vascular risk factor. The mean number of volumetric MRI measures over time was 3 (max = 18), with a mean 5.3 years between the first and last MRI scan (max = 24 years). Out of the 1541 participants with volumetric data, 1348 also had one or more WMH measure (mean number of measures over time = 2, max = 10; mean time between baseline and last WMH measure = 2.9 years, max = 19 years).

**Table 1:** Participant characteristics at baseline

	Participants in *APOE* analyses with volumetric data	Participants in *APOE* analyses with WMH data	Participants in AD-PRS analyses with volumetric data	Participants in AD-PRS analyses with WMH data
*N*	1541	1348	1093	972
Age at baseline MRI scan, M (SD)	66.2 (9.6)	68.7 (9.6)	66.1 (9.4)	68.6 (9.6)
Female sex, *N* (%)	929 (60.3%)	816 (60.6%)	672 (61.5)	605 (62.2%)
Years of education, M (SD)	15.2 (3.1)	15.2 (3.2)	15.0 (3.2)	15.1 (3.2)
Race, White, *N* (%)	1449 (94.0%)	1273 (94.4%)	1093 (100%)	972 (100%)
MMSE score, M (SD)	29.0 (1.2)	29.0 (1.3)	29.1 (1.1)	29.1 (1.3)
Progressed to MCI/dementia, *N* (%)	94 (6.1%)	82 (6.1%)	71 (6.5%)	64 (6.6%)
Vascular risk score, M (SD)	1.1 (1.0)	1.1 (1.1)	1.1 (1.0)	1.1 (1.0)
Vascular risk score ≥ 1, *N* (%)	1038 (67.4%)	904 (67.2%)	727 (66.5%)	648 (66.7%)
Vascular risk score ≥ 2, *N* (%)	481 (31.2%)	433 (32.2%)	326 (29.8%)	307 (31.6%)
Vascular risk score ≥ 3, *N* (%)	164 (10.5%)	155 (11.5%)	98 (9.0%)	101 (10.4%)
Genetic factors				
*APOE* ɛ2 carriers, *N* (%)^a^	184 (11.9%)	164 (12.2%)	125 (11.5%)	113 (11.6%)
*APOE* ɛ4 carriers, *N* (%)^b^	495 (32.1%)	417 (31.0%)	339 (31.0%)	293 (30.1%)
*APOE* ɛ3/ɛ3 carriers, *N* (%)	862 (55.9%)	765 (56.8%)	629 (57.5%)	566 (58.2%)
*APOE* ɛ4/ɛ4 carriers, *N* (%)	63 (4.1%)	52 (3.9%)	46 (4.2%)	37 (3.8%)
*APOE* ɛ3/ɛ4 carriers, *N* (%)	385 (25.5%)	328 (24.4%)	263 (24.1%)	232 (23.9%)
*APOE* ɛ2/ɛ4 carriers, *N* (%)	46 (3.0%)	37 (2.7%)	30 (2.7%)	24 (2.5%)
AD-PRS, M (SD)			−0.02 (0.97)	−0.02 (0.97)
Baseline MRI measures				
SPARE_AD, M (SD)	−1.3 (0.8)	−1.2 (0.9)	−1.3 (0.8)	−1.2 (0.9)
SPARE_BA, M (SD)	66.8 (11.2)	68.6 (11.4)	66.7 (10.9)	68.6 (11.3)
SPARE_BA residual, M (SD)	−0.3 (7.1)	−0.1 (7.2)	−0.3 (7.1)	−0.0 (7.1)
Hippocampal volume, M (SD) in mm^3^	3766 (404)	3734 (404)	3768 (402)	3730 (398)
WMH volume (in mm^3^), M (SD)	2810 (5408)	3105 (5837)	2821 (5540)	3200 (6096)
Number of MRI measures over time, M (SD) [range]	3.0 (2.5) [1–18]	2.0 (1.2) [1–10]	3.1 (2.5) [1–18]	2.0 (1.2) [1–8]
*N* participants with two or more MRI scans over time, (%)	998 (64.8%)	769 (57.1%)	777 (71.1%)	599 (61.6%)
Years between baseline and last MRI, M (SD) [range]	5.3 (5.7) [0–24.4]	2.9 (4.0) [0–18.9]	5.7 (5.7) [0–24.4]	3.0 (3.9) [0–18.9]

^a^Includes ɛ2/ɛ2 and ɛ2/ɛ3 carriers.

^b^Includes ɛ2/ɛ4, ɛ3/ɛ4 and ɛ4/ɛ4 carriers.

### *APOE* genotypes and MRI trajectories

Results from the primary model examining the binary *APOE*-ɛ2 and ɛ4 indicators in relationship to trajectories of the MRI measures are shown in Table 2 (with ɛ3/ɛ3 as the reference). *APOE*-ɛ4 carrier status was not associated with any MRI measure at baseline (all *P* ≥ 0.14). However, relative to ɛ3/ɛ3 carriers, ɛ4 carriers demonstrated greater increases in SPARE-AD (*P* < 0.001) scores and greater decreases in hippocampal volume (*P* ≤ 0.001) over time; they also showed greater increases in SPARE-BA (*P* = 0.025), though the effect appeared smaller (*Z* = 2.25 versus *Z* = 3.45 for SPARE-AD; see Supplementary Text 2 for formal model comparison). *APOE*-ɛ2 carrier status was also not associated with any baseline MRI measure (all *P* ≥ 0.12), but ɛ2 carriers showed greater increases in WMH volumes over time (*P* = 0.014), compared to ɛ3/ɛ3 carriers. There was no association between ɛ2 carrier status and rate of change in the other MRI measures (all *P* > 0.3).

**Table 2:** Mixed effects model results of APOE genetic status in relationship to MRI measures

	SPARE-AD (AD-related atrophy)		SPARE-BA-resid (age-related atrophy)		Hippocampus volume		WMH volume	
Model predictors	Estimate (SE)	*P*-value	Estimate (SE)	*P*-value	Estimate (SE)	*P*-value	Estimate (SE)	*P*-value
Time	−0.184 (0.025)	<0.0001	−0.037 (0.021)	0.09	0.104 (0.018)	<0.0001	−0.102 (−0.023)	<0.0001
Time^2^	0.003 (0.0003)	<0.0001	−0.001 (0.0003)	<0.0001	−0.0004 (0.0002)	0.038		
Age	0.038 (0.003)	<0.0001	−0.012 (0.003)	0.001	−0.035 (0.003)	<0.0001	0.051 (0.003)	<0.0001
Age × time	0.003 (0.0004)	<0.0001	0.001 (0.0003)	<0.0001	−0.003 (0.0003)	<0.0001	0.002 (0.0003)	<0.0001
Sex (female)	−0.002 (0.049)	0.97	0.013 (0.053)	0.80	−0.052 (0.048)	0.29	0.022 (0.050)	0.66
Sex (F) × time	−0.016 (0.006)	0.009	−0.028 (0.005)	<0.0001	0.007 (0.004)	0.08	0.007 (0.005)	0.20
Education	0.008 (0.025)	0.74	0.029 (0.026)	0.27	0.014 (0.024)	0.56	−0.001 (0.025)	0.97
Education × time	0.008 (0.003)	0.74	−0.003 (0.002)	0.17	−0.001 (0.002)	0.75	−0.001 (0.003)	0.66
*APOE*-*ɛ2*	−0.042 (0.066)	0.53	0.018 (0.071)	0.80	0.009 (0.065)	0.89	0.008 (0.067)	0.12
*APOE*-*ɛ2* × time	0.002 (0.008)	0.84	−0.000 (0.006)	0.99	0.005 (0.005)	0.34	0.016 (0.007)	0.014
*APOE*-*ɛ4*	0.076 (0.051)	0.14	−0.045 (0.055)	0.41	−0.010 (0.050)	0.83	−0.042 (0.052)	0.42
*APOE*-*ɛ4* × time	0.021 (0.006)	0.001	0.011 (0.005)	0.025	−0.167 (0.004)	<0.0001	0.008 (0.005)	0.13

Additionally, across all models (Table 2), older age was associated with higher SPARE-AD scores, smaller hippocampal volumes and greater WMH volumes at baseline and greater increases in SPARE-AD, SPARE-BA scores and WMH volumes over time, as well as greater decreases in hippocampal volume over time (all *P* < 0.0001). Participant sex was not associated with any baseline MRI measure, but females had smaller increases in SPARE-AD (*P* = 0.009) and SPARE-BA (*P* < 0.0001) scores over time than males. Years of education was not associated with the baseline or rate of change in any MRI measure (all *P* ≥ 0.17). These results remained the same when excluding *APOE*-*ɛ2*/*ɛ4* carriers from the analysis (data not shown), except that the association between *APOE*-ɛ4 carrier status and rate of change in SPARE-BA was no longer significant (estimate = 0.010, SE = 0.005, *P* = 0.069).

The pattern of results was also the same when additionally adjusting for vascular risk scores and follow-up diagnosis (see Supplementary Table 4). In these models, higher vascular risk scores were associated with higher baseline SPARE-AD scores (*P* = 0.026), but were not related to the rate of change in any MRI measure (all *P* ≥ 0.05). Additionally, participants who progressed to MCI/dementia over time had higher baseline WMH volumes (*P* = 0.012), greater increases in WMH volumes (*P* = 0.012) and SPARE-AD scores (*P* < 0.0001) over time, and greater declines in hippocampal volume (*P* < 0.0001) after also adjusting for vascular risk and follow-up diagnosis.

**Figure 1:**
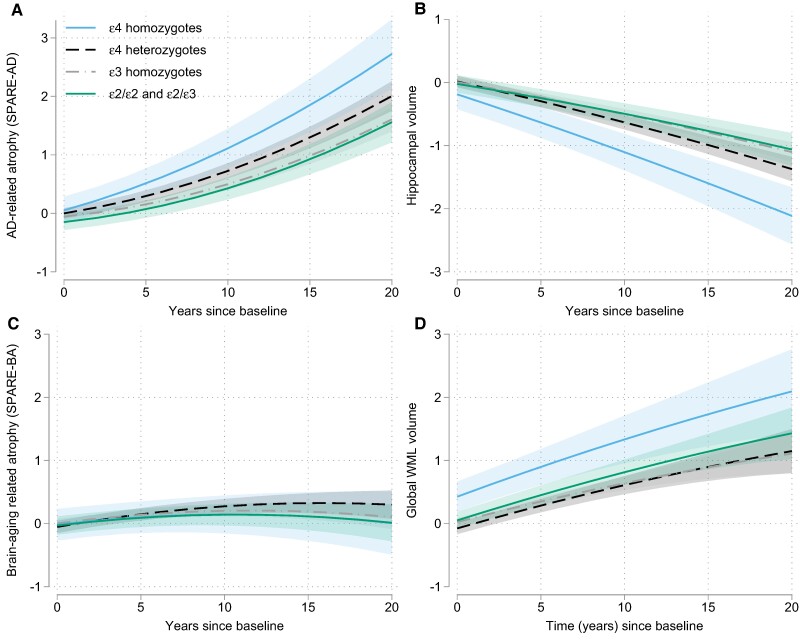
**Longitudinal volumetric atrophy and WMH volumes as a function of APOE genetic status.** Figure shows estimates from mixed effects regression model examining *APOE* genetic status in relation to (**A**) AD-related atrophy, measured by SPARE-AD scores, (**B**) hippocampal volumes, and (**C**) age-related atrophy, and (**D**) WMH volumes. The rate of change in AD-related atrophy (estimate = 0.033, SE = 0.015, *P* = 0.028) and hippocampal volumes (estimate = 0.026, SE = 0.011, *P* = 0.014) was greater for individuals with two *ɛ4* alleles compared to those with one ɛ*4* allele, who in turn showed more atrophy than *ɛ3*/*ɛ3* carriers (estimate = 0.017, SE = 0.006, *P* = 0.008 for SPARE = AD and (estimate = 0.013, SE = 0.004, *P* = 0.003 for hippocampus). Atrophy rates did not differ between ɛ2 carrier and *ɛ3*/*ɛ3* carriers (all *P* > 0.3). WMH volumes (**D**) increased more over time among both *ɛ4*/*ɛ4* carriers (estimate = 0.029, SE = 0.014, *P* = 0.039) and *ɛ2* carriers (estimate = 0.015, SE = 0.008, *P* = 0.049) relative to *ɛ3*/*ɛ3* carriers (see text for details).

**Figure 2:**
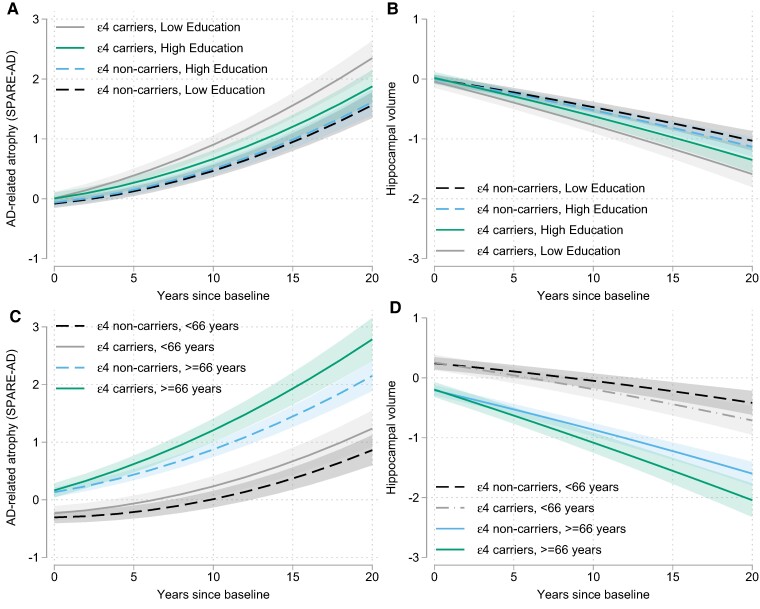
**Longitudinal volumetric atrophy based on APOE-ɛ4 genetic status and participant education and age.** Estimates from mixed effects regression model showing how years of education (**A** and **B**) and baseline age (**C** and **D**) modify the association between *APOE*-ɛ4 genetic status and rate of change of Alzheimer's disease-vulnerable regions, measured by SPARE-AD scores (**A** and **C**), and the hippocampus (**B** and **D**). The negative effect of *APOE*-ɛ4 genetic status on rate of atrophy in Alzheimer's disease-vulnerable regions and the hippocampus was greater among older than young participants (**C** and **D**), as indicated by significant three-way interactions of *ɛ4* × age × time for SPARE-AD (estimate = 0.002, SE = 0.001, *P* = 0.001) and for the hippocampus (estimate = −0.001, SE = 0.0004, *P* = 0.027), but was attenuated among individuals with more years of education (**A** and **B**), as indicated by significant *ɛ4* × education × time interactions for SPARE-AD (estimate = −0.015, SE = 0.006, *P* = 0.017) and for the hippocampus (estimate = 0.009, SE = 0.004, *P* = 0.039). For illustration purposes, the 25th and 75th percentiles of the baseline education were used to show trajectories of high versus low education.

When modelling *APOE* as a categorical variable, the pattern of results was the same. Additionally, we observed that SPARE-AD scores increased more among *APOE* ɛ4/ɛ4 carriers relative to ɛ4 heterozygous participants (estimate = 0.033, SE = 0.015, *P* = 0.028), who had greater increases than ɛ3/ɛ3 carriers (estimate = 0.017, SE = 0.006, *P* = 0.008). This is illustrated in Fig. 1A. A similar pattern was observed for hippocampal volumes, which showed greater decline over time among ɛ4/ɛ4 carriers compared to ɛ4 heterozygous individuals (estimate = 0.026, SE = 0.011, *P* = 0.014), who showed greater decline relative to ɛ3/ɛ3 carriers (estimate = 0.013, SE = 0.004, *P* = 0.003), see Fig. 1B. There was no difference in the SPARE-BA trajectories between individuals with one versus two *ɛ4* alleles (*P* > 0.05; Fig. 1C). For WMH volumes, both *ɛ4*/*ɛ4* carriers (estimate = 0.029, SE = 0.014, *P* = 0.039) and *ɛ2* carriers (*ɛ2*/*ɛ2* and *ɛ2*/*ɛ3* combined, estimate = 0.015, SE = 0.008, *P* = 0.049) showed greater increases over time than *ɛ3*/*ɛ3* carriers, who did not differ from *ɛ4* heterozygous participants (estimate = 0.007, SE = 0.006, *P* = 0.23), Fig. 2D. Differences between ɛ4 homozygous and heterozygous individuals remained the same when additionally covarying follow-up diagnosis and vascular risk scores (data not shown).

Results from models examining whether the demographic and clinical variables modified the relationships between *APOE* genotypes and the MRI trajectories are shown in Table 3. The associations of *APOE*-*ɛ4* genetic status with rates of change in SPARE-AD scores and hippocampal volume were modified by baseline age and years of education (all *P* for interaction terms of *ɛ4* × (age or education) × time ≤ 0.039). Specifically, *ɛ4* related atrophy in SPARE-AD regions and the hippocampus was greater among older participants and weaker among those with more years of education. These three-way interactions remained significant when excluding *APOE ɛ2*/*ɛ4* carriers (all *P* ≤ 0.012) and are illustrated in Fig. 2. Additionally, the relationship between *ɛ4* genetic status and the rate of decline in hippocampal volume was greater among participants with higher compared to lower vascular risk scores (*P* = 0.038) and among those who progressed to MCI or dementia over time compared to those who remained cognitively unimpaired (*P* = 0.001), see Table 3. However, the three-way interactions with vascular risk scores or progressor status were not significant when excluding *APOE ɛ2*/*ɛ4* carriers (both *P* > 0.21). Among *APOE*-*ɛ2* carriers, higher education was unexpectedly associated with greater increases in SPARE-AD over time (*P* = 0.032); however, this interaction was also not significant after exclusion of *APOE ɛ2*/*ɛ4* carriers (*P* > 0.15).

**Table 3:** Results from mixed effects models testing whether associations between genetic risk factors and rate of change in MRI measures differ by demographic and clinical variables

	SPARE-AD (AD-related atrophy)		SPARE-BA-resid (age-related atrophy)		Hippocampus volume		WMH volume	
	Estimate (SE)	*P*-value	Estimate (SE)	*P*-value	Estimate (SE)	*P*-value	Estimate (SE)	*P*-value
Age								
*APOE*-*ɛ2* × age × time	−0.001 (0.001)	0.36	−0.001 (0.001)	0.07	0.001 (0.001)	0.10	−0.000 (0.001)	0.96
*APOE*-*4* × age × time	**0.002** (**0.001)**	**0**.**001**	0.000 (0.001)	0.69	**−0.001** (**0.000)**	**0**.**027**	−0.000 (0.001)	0.67
AD-PRS × age × time	−0.000 (0.001)	0.63	−0.000 (0.000)	0.15	0.000 (0.000)	0.45	0.000 (0.000)	0.11
Sex								
*APOE*-*ɛ2* × sex × time	−0.005 (0.016)	0.78	−0.008 (0.014)	0.57	−0.004 (0.011)	0.69	−0.016 (0.014)	0.27
*APOE*-*4* × sex × time	−0.015 (0.013)	0.26	−0.005 (0.011)	0.63	−0.000 (0.009)	0.97	−0.002 (0.012)	0.89
AD-PRS × sex × time	0.008 (0.007)	0.24	−0.002 (0.005)	0.78	−0.005 (0.005)	0.26	−0.001 (0.006)	0.88
Education								
*APOE*-*ɛ2* × Educ × time	**0.018** (**0.008)**	**0**.**032**	0.005 (0.007)	0.49	−0.010 (0.006)	0.08	−0.005 (0.008)	0.48
*APOE*-*ɛ4* × Educ × time	**−0.015** (**0.006)**	**0**.**017**	−0.007 (0.006)	0.20	**0.009** (**0.004)**	**0**.**039**	−0.006 (0.006)	0.28
AD-PRS × Educ × time	0.007 (0.004)	0.058	0.002 (0.003)	0.51	−0.004 (0.002)	0.10	−0.000 (0.003)	0.99
Vascular risk scores (VRS)								
*APOE*-*ɛ2* × VRS × time	0.005 (0.005)	0.29	−0.002 (0.005)	0.69	0.001 (0.004)	0.77	0.006 (0.006)	0.33
*APOE* ɛ4 × VRS × time	0.002 (0.004)	0.59	0.002 (0.004)	0.58	**−0.006** (**0.003)**	**0**.**038**	−0.042 (0.025)	0.09
AD-PRS × VRS × time	0.003 (0.002)	0.20	−0.002 (0.002)	0.29	−0.002 (0.002)	0.23	0.003 (0.003)	0.30
Progressed								
*APOE*-*ɛ2* × Progr × time	0.028 (0.026)	0.29	−0.019 (0.023)	0.42	0.011 (0.018)	0.54	0.004 (0.030)	0.89
*APOE*-*ɛ4* × Progr × time	0.022 (0.019)	0.25	−0.008 (0.017)	0.65	**−0.046** (**0.013)**	**0**.**001**	−0.011 (0.019)	0.55
AD-PRS × Progr × time	−0.004 (0.010)	0.66	−0.005 (0.008)	0.51	0.012 (0.007)	0.09	−0.002 (0.009)	0.80

Three-way interaction terms for each demographic variable (i.e. baseline age, sex and years of education) or clinical variable (i.e. VRS score and progressed status) with *APOE*-ɛ4 and *APOE*-ɛ2 (or AD-PRS scores) and time were tested in separate models, adjusting for baseline age, sex, years of education, cohort indicators and interactions of all predictors with time. All lower-order interaction terms for the three-way interactions were also included. Bold values represent significant effects at *P* < 0.05.

### Alzheimer's disease-polygenic risk score and MRI trajectories

**Figure 3:**
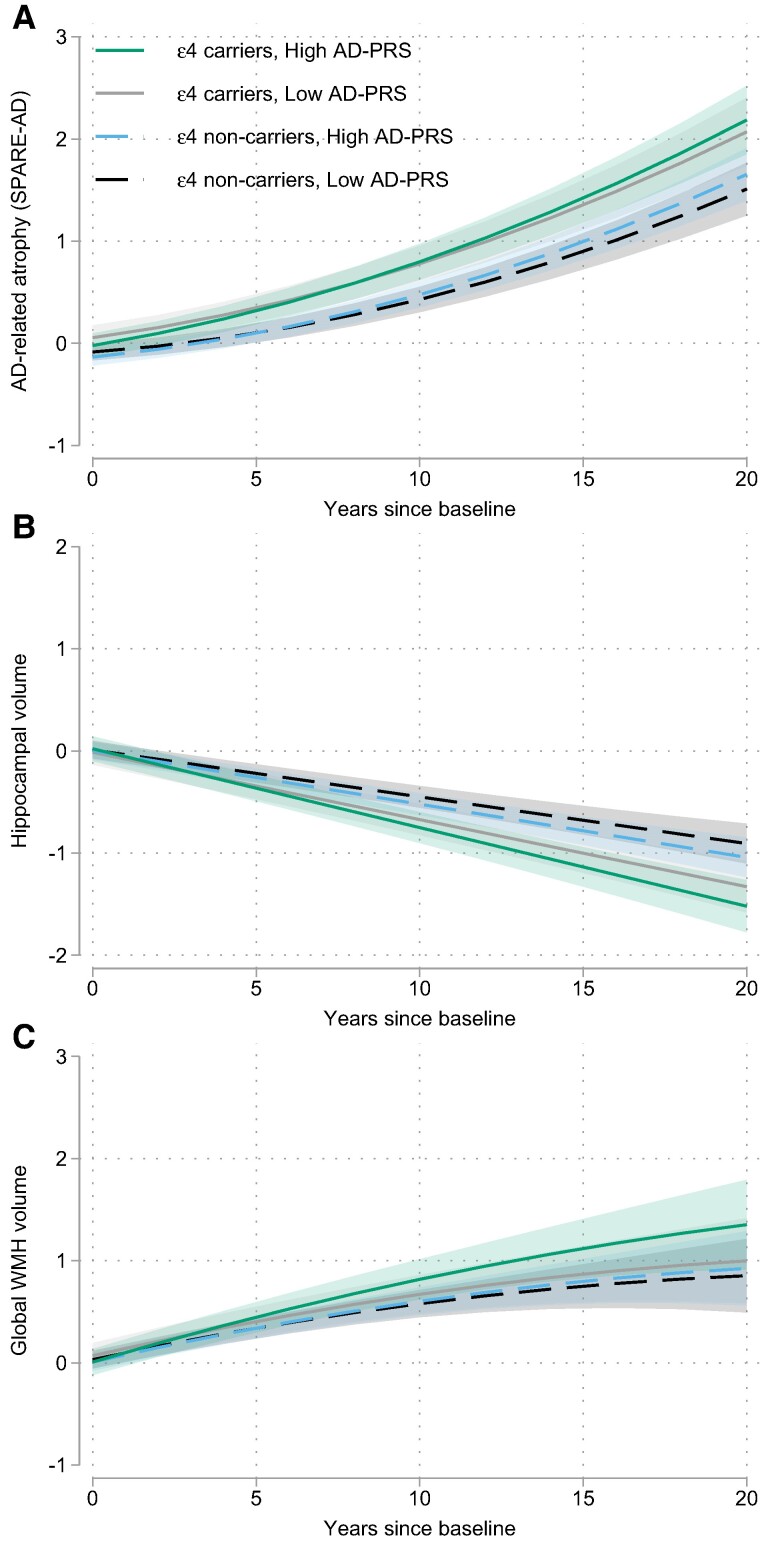
**Longitudinal volumetric atrophy and WMH volumes as a function of AD-polygenic risk and *APOE*-*ɛ4*.** Estimates from mixed effects regression model showing independent associations between AD-polygenic risk scores and *APOE*-ɛ4 genetic status with longitudinal atrophy in AD-vulnerable regions (i.e. SPARE-AD scores, **A**, estimate = 0.007, SE = 0.003, *P* = 0.035) and the hippocampus (**B**, estimate = −0.006, SE = 0.003, *P* = 0.014). For global WMH volumes (**C**), *APOE*-ɛ4 genetic status was more strongly associated with increases in WMH volumes among those with higher compared to lower AD-polygenic risk scores (AD-PRS × *APOE*-ɛ4 × time interaction, estimate = 0.014, SE = 0.007, *P* = 0.038). For illustration purposes, the 25th and 75th percentiles of the baseline AD-PRS scores were used to show trajectories of high versus low PRS.

In the primary models, there was no association between AD-PRS scores and baseline levels of the MRI measures (all *P* ≥ 0.12). By contrast, higher AD-PRS scores were associated with greater increases over time in SPARE-AD scores, greater decreases in hippocampal volume and greater increases in WMH volumes (all *P* ≤ 0.035, see Table 4 and Fig. 3). Results were similar using a dichotomous AD-PRS (see Supplementary Table 5). The associations between the AD-PRS score and rate of change in the MRI measures were independent of *APOE* genetic status and were not modified by age, sex, years of education, vascular risk scores or progressor status (see Table 3).

**Table 4:** Mixed effects model results of AD-polygenic risk score and *APOE* genetic status in relationship to MRI measures

	SPARE-AD (AD-related atrophy)		SPARE-BA-resid (age-related atrophy)		Hippocampus volume		WMH volume	
	Estimate (SE)	*P*-value	Estimate (SE)	*P*-value	Estimate (SE)	*P*-value	Estimate (SE)	*P*-value
AD-PRS	−0.045 (0.029)	0.12	−0.001 (0.032)	0.97	0.006 (0.030)	0.85	−0.028 (0.031)	0.38
AD-PRS × time	0.007 (0.003)	0.035	0.004 (0.003)	0.15	−0.006 (0.003)	0.014	0.008 (0.003)	0.009
*APOE*-*ɛ2*	0.061 (0.079)	0.44	0.061 (0.086)	0.48	−0.093 (0.081)	0.25	0.069 (0.085)	0.42
*APOE*-*ɛ2* × time	−0.000 (0.010)	0.99	−0.004 (0.008)	0.65	0.006 (0.007)	0.36	0.006 (0.008)	0.43
*APOE*-*ɛ4*	0.131 (0.061)	0.032	−0.049 (0.066)	0.46	−0.009 (0.062)	0.89	0.025 (0.065)	0.70
*APOE*-*ɛ4* × time	0.021 (0.007)	0.003	0.010 (0.006)	0.09	−0.022 (0.005)	<0.0001	0.014 (0.006)	0.027

All models were adjusted by baseline age, sex, years of education, indicators for each cohort and included interactions of each predictor with time (e.g. terms for all genetic predictors × time and covariates × time).

Lastly, we explored potential interactions between the AD-PRS scores and *APOE*-*ɛ4* and *ɛ2* genetic status in relationship to change in the MRI measures. To simplify interpretation, *APOE*-*ɛ2*/ɛ4 carriers were excluded from these analyses. There were no interactions between the AD-PRS score and APOE-*ɛ4* or *APOE*-*ɛ2* genetic status with respect to the rate of change in SPARE-AD, SPARE-BA and hippocampal volume (all *P* > 0.47). However, for WMH volumes, there was an AD-PRS × *APOE*-*ɛ4* × time interaction (*P* = 0.038, see Supplementary Table 6), suggesting a stronger association between *APOE*-*ɛ4* genetic status and rate of increase in WMH among participants with higher compared to lower AD-PRS scores (Fig. 3C).

## Discussion

The large sample size and substantial follow-up period of the current study provide the basis for several new insights on the relationship between genetic risk factors for late-onset Alzheimer's disease among cognitively normal individuals and changes in MRI measures of brain atrophy and WMH. First, the atrophy rates in a composite volume measure of Alzheimer's disease-vulnerable regions (SPARE-AD) and the hippocampus demonstrated an *APOE*-*ɛ*4 gene-dose effect, with greatest atrophy among *ɛ*4 homozygous participants, followed by *ɛ*4 heterozygous participants, and least among *ɛ*4 non-carriers. Second, both *APOE*-*ɛ*4 status and AD-PRS scores independently influenced rates of change in AD-vulnerable regions and the hippocampus, suggesting additive effects. Third, the negative impact of *APOE*-*ɛ*4 on atrophy in AD-vulnerable regions and the hippocampus was reduced among individuals with higher education and younger baseline ages. Fourth, *ɛ*4 carrier status was associated with greater increases in global WMH volumes over time, particularly among *ɛ*4 homozygous participants and those with high Alzheimer's diesease-polygenic risk scores. Fifth, *ɛ*2 carrier status did not influence atrophy rates in regions sensitive to aging or Alzheimer's disease, but was associated with greater increases in WMH volumes over time. In contrast, neither *APOE* nor AD-PRS scores showed robust associations with atrophy in a composite measure of regions sensitive advanced non-Alzheimer's disease-related brain aging (SPARE-BA), supporting prior evidence that this measure largely reflects age but not disease-related atrophy. Taken together, these results underscore the impact of AD-genetic risk factors on rates of change in MRI measures of neurodegeneration among middle-aged and older adults with normal cognition and point to potential interactions and synergistic effects between *APOE* and other Alzheimer's disease risk genes on WMH burden.

### *APOE*-e4 genetic status and brain atrophy

The current results are consistent with prior longitudinal studies among participants across the clinical spectrum of Alzheimer's disease^33^ and non-demented cohorts^30-32^ that have reported elevated longitudinal atrophy in Alzheimer's disease-vulnerable regions among *ɛ*4 carriers compared to non-carriers. Our findings are also in line with, and extend prior work, among individuals with normal cognition^62,63,97^ by documenting an *APOE*-*ɛ*4 gene-dose effect on atrophy rates in the hippocampus and a composite of Alzheimer's disease-vulnerable regions. This gene-dose effect likely reflects the fact that Alzheimer's disease pathology (i.e. amyloid and tau) begins to accumulate at an earlier age among *ɛ*4 carriers compared to non-carriers, with *ɛ*4 homozygous individuals showing the youngest age of onset of amyloid accumulation and amyloid positivity, followed *ɛ*4 heterozygous individuals, and then *ɛ*4 non-carriers.^98-100^ It is hypothesized that this earlier age of amyloid accumulation likely initiates an earlier onset of AD-related atrophy in selected brain regions. This is consistent with the view that subtle Alzheimer's disease-related atrophy begins during the preclinical phase of the disease, when individuals are cognitively normal.^8,9,101^ The lack of volumetric differences between *ɛ*4 carriers and non-carriers at baseline supports the view that *APOE*-*ɛ*4 primarily influences brain volumes during the preclinical phase of Alzheimer's disease, but has limited impact on volumes prior to midlife, though such differences have been demonstrated.^26,102^ Our results also demonstrate that *APOE*-*ɛ*4 related differences in hippocampal atrophy appear to be particularly evident among individuals who progress to MCI or dementia over time, in line with a prior study.^97^ The finding that *ɛ*4 was only weakly associated with atrophy in non-Alzheimer's disease regions that are sensitive to aging (SPARE-BA) underscores the specificity of *ɛ*4 to Alzheimer's disease-related atrophy and might reflect the fact that in some individuals, Alzheimer's disease pathology begins in more atypical regions.^103^

Our results also showed that the association between *APOE*-*ɛ*4 genetic status and rate of atrophy in the hippocampus and the Alzheimer's disease-vulnerable regions increases with advancing age (Fig. 2C and D), in line with the age-related increase in Alzheimer's disease pathology accumulation^98^ and the age-related increase in Alzheimer's disease-related cognitive impairment. This finding might also explain why some prior studies among middle-aged cohorts have failed to find cross-sectional volumetric differences by *ɛ*4-status.^56-58^ We found no evidence that the association between *APOE*-*ɛ*4 and atrophy in AD-vulnerable regions or regions sensitive to aging (SPARE-BA) was influenced by overall levels of vascular risk, though for the hippocampus, higher vascular risk scores were associated with a stronger relationship between *APOE*-*ɛ*4 genetic status and atrophy over time (but this was not significant when excluding *ɛ*2/*ɛ*4 carriers). Although vascular risk factors have been consistently linked with smaller regional brain volumes^28,104,105^ and atrophy rates,^47,97^ little is known about whether *APOE* variants moderate this relationship. Additionally, the relationship between vascular risk and brain atrophy may differ by other factors, such as amyloid burden^106^ and by type of vascular risk factor (e.g. hypertension versus obesity).^64^ Thus, summary scores using different risk factors, or differentially weighted risk factors, may potentially show stronger associations with brain atrophy than was observed here.

In this study, we also observed that years of education modified the association between *APOE*-*ɛ*4 genetic status and atrophy in the Alzheimer's disease-vulnerable regions composite and the hippocampus, such that participants with more years of education had less *ɛ*4-related atrophy than those with less education. These findings are in line with a recent study also using data from the PAC cohort (*N* = 1819), which reported that higher scores on a composite measure of years education and literacy attenuated the negative effect of *APOE*-*ɛ*4 genotype on the rate of decline in episodic memory and a global cognitive score.^74^ The present results suggest that this reduction in *APOE*-*ɛ*4 related cognitive decline among participants with more education may be mediated by reduced atrophy in Alzheimer's disease-vulnerable regions. Previous cross-sectional studies have produced mixed results regarding the association of years of education and regional brain volumes among middle-aged and older cognitively unimpaired individuals (e.g. Launer *et al*.,^107^ Arenaza-Urquijo *et al*.,^108^ Liu *et al*.^109^ and Vemuri *et al*.^110^). Among the few prior longitudinal studies, most found no association between education or related measures of literacy and change in brain volumes or cortical thickness over time.^8,111-113^ The present results suggest that some inconsistencies across prior studies may be attributable to the fact that the education-related reduction in atrophy is relatively small and primarily evident among *APOE*-*ɛ*4 carriers, making it difficult to detect in studies with smaller samples.

### *APOE*-*ɛ*2 genetic status and brain atrophy

Another important finding is that *APOE*-*ɛ*2 carriers demonstrated similar rates of brain atrophy over time as *ɛ*3 homozygous individuals and did not differ in terms of brain volumes at baseline. Given the relatively low prevalence of the *ɛ*2 allele, prior work on this subject has been limited and largely comprised of cross-sectional studies with small numbers of *ɛ*2 carriers (ranging from ∼12 to 85 compared to 184 in this study). Our results are consistent with two prior cross-sectional studies among cognitively normal individuals that also found no difference in volumetric measures as a function of *ɛ*2 carrier status among middle-aged and older adults.^54,61^ By comparison, a small-scale longitudinal study^66^ and a few other cross-sectional studies^52,60,114^ reported reduced 2-year atrophy and greater cortical thickness and volumes among older cognitively normal *ɛ*2 carriers compared to *ɛ*3 homozygotes in regions sensitive to Alzheimer's disease. A likely explanation for these discrepancies across studies is that *ɛ*2 carriers are less likely to harbour preclinical Alzheimer's disease pathology (due to a later age of amyloid accumulation). Consequently, they are less likely to have atrophy in Alzheimer's disease-sensitive regions during middle- and old age compared to *ɛ*3/*ɛ*3 carriers, which can appear as reduced atrophy or greater volume. Future studies will be able to test this possibility by covarying amyloid and tau burden when evaluating associations between *APOE*-*ɛ*2 status and atrophy.

### *APOE*-*ɛ*2 and *ɛ*4 genetic status and white matter hyperintensities

Prior studies among non-demented participants as well as samples spanning the Alzheimer's disease-spectrum have reported higher WMH burden^37-40^ and greater longitudinal increases in WMH burden^42,43^ among *APOE*-*ɛ*4 carriers relative to carriers, with stronger associations for homozygous than heterozygous participants^38^ (but see Habes *et al*.,^44^ Lyall *et al*.,^45^ Lane *et al*.^46^ and Debette *et al*.^47^ for negative results). Few studies, however, have examined *ɛ*4-related differences in WMH volumes among individuals with normal cognition.^48,49,115^ The present study found greater longitudinal increases in global WMH volumes among *ɛ*4 homozygous compared to *ɛ*4 heterozygous participants and *ɛ*3/*ɛ*3 carriers, who did not differ from one another. These findings are consistent with a cross-sectional study among cognitively normal middle-aged participants.^49^ Given that cognitively unimpaired middle-aged and older *ɛ*4/*ɛ*4 carriers likely harbour the highest level of brain amyloid,^98^ these findings support the view that Alzheimer's disease-specific pathways contribute to the formation of WMH among individuals with normal cognition.^4,5,116^ This contribution may be subtle during the preclinical phase of AD, when pathology levels are low, and increase as the disease progresses, as evidenced by more robust associations of WMH burden with *ɛ*4 genetic status^37,43^ or Alzheimer's disease-biomarker levels^116-118^ among symptomatic individuals. Our finding of an interaction between *APOE*-*ɛ*4 and AD-PRS scores in relation to WMH trajectories further suggests that associations between *APOE*-*ɛ*4 and WMH load may be more evident among those with additional Alzheimer's disease risk genes, beyond *APOE*.

The current study is the first, to our knowledge, to demonstrate greater longitudinal increases in WMH burden among cognitively unimpaired *APOE*-*ɛ*2 carriers relative to *ɛ*3/*ɛ*3 carriers. This finding is consistent with and expands prior cross-sectional studies among non-demented and cognitively impaired cohorts that have also reported *ɛ*2-related elevations in WMH burden.^40,119^ Two cross-sectional studies among cognitively normal middle-aged participants found no *ɛ*2-related WMH differences,^49,120^ consistent with the absence of a baseline difference in WMH volumes by *ɛ*2 genetic status in this study. Altogether, these results support the view that the *APOE*-*ɛ*2 allele promotes cerebrovascular disease, though the mechanisms remain poorly understood.^121^

### Alzheimer's disease-polygenic risk, brain atrophy and WMH burden

Another important finding of this study was that higher AD-PRS scores were associated with greater atrophy over time in Alzheimer's disease-vulnerable regions, including the hippocampus, independent of *APOE*-*ɛ*4 status. This extends prior findings from longitudinal studies with participants across the Alzheimer's disease-spectrum^18,34,35^ and from cross-sectional studies among non-demented corhorts^21,23,24,28,29^ and suggest that higher Alzheimer's disease-polygenic risk scores increase the risk of neurodegeneration not only during the symptomatic phase of the disease but also among participants with normal cognition. Furthermore, AD-PRS-related atrophy was independent of age, sex, education and vascular risk scores, and not evident for regions that show non-Alzheimer's disease-related atrophy with age, consistent with a cross-sectional study.^28^ This suggests that *APOE*-*ɛ*4 and other AD risk genes influence atrophy in common Alzheimer's disease-susceptible brain regions, including the hippocampus, as early as midlife.

Higher AD-PRS were also associated with greater increases in global WMHs over time, particularly among *APOE*-*ɛ*4 carriers. This suggests that other Alzheimer's disease risk genes may exert some of their effects via cerebrovascular mechanisms, in addition to Alzheimer's disease-specific pathways. This interpretation is consistent with evidence linking higher AD-PRS scores to reductions in cerebral blood flow^122^ and neuropathological markers of cerebrovascular disease,^34^ though results might differ for PRS scores computed using other methodologies. Future studies that also include AD-biomarker assessments of amyloid and tau are needed to clarify how AD-PRS influence neurodegeneration.

## Conclusion

The generalizability of findings from the current study to the broader population is limited because participants were primarily White, well-educated and enriched for a family history of Alzheimer's disease-dementia. For example, atrophy is more likely due to Alzheimer's disease in the current study than in the general population, and we may have overestimated associations between Alzheimer's disease-genetic risk with atrophy and underestimated other factors, like vascular risk. Additionally, these analyses do not include biomarkers of Alzheimer's disease pathology or other measures of brain structure and function, which precludes inferences regarding the precise mechanisms by which Alzheimer's disease-genetic risk influences brain atrophy among unimpaired individuals. Also, the power to detect significant three-way interactions involving Alzheimer's disease-genetic variables for the observed effects was only low to moderate. Nonetheless, the study provides compelling evidence that *APOE*-*ɛ*4 and AD-PRS independently and additively influence longitudinal trajectories of neurodegeneration in Alzheimer's disease-sensitive regions and synergistically increase WMH accumulation among cognitively normal individuals. Conversely, *APOE*-*ɛ*2 primarily influences WMH accumulation, but not atrophy. These AD-genetic associations did not differ by participant sex, but in some cases were influenced by participant age, years of education and vascular risk, providing potential avenues for reducing the negative impact of AD risk genes on neurodegeneration prior to the development of cognitive impairment. Future studies are needed to examine the degree to which these Alzheimer's disease-genetic-related brain changes mediate changes in cognition.

## Supplementary Material

fcae276_Supplementary_Data

## Data Availability

The plan is to archive the PAC datafiles at the National Archive of Computerized Data on Aging (NACDA). Investigators interested in accessing the data should contact the PAC Coordinating Center at Johns Hopkins University for details.
