# Supplementary material for: Alzheimer’s disease genetic risk and changes in brain atrophy and white matter hyperintensities in cognitively unimpaired adults
Source: Brain Commun. 2024 Aug 14;6(5):fcae276. doi: 10.1093/braincomms/fcae276 (PMC11369827; doi:10.1093/braincomms/fcae276)
Supplement: fcae276_Supplementary_Data [file fcae276_supplementary_data.pdf]

Supplementary Materials

Supplementary Figure 1

Masks showing the SPARE-AD and SPARE-BA composite regions. Colored regions represent positively weighted (W) regions in the mask, with regions with higher weights (i.e., more strongly weighted in the pattern) shown in yellow.

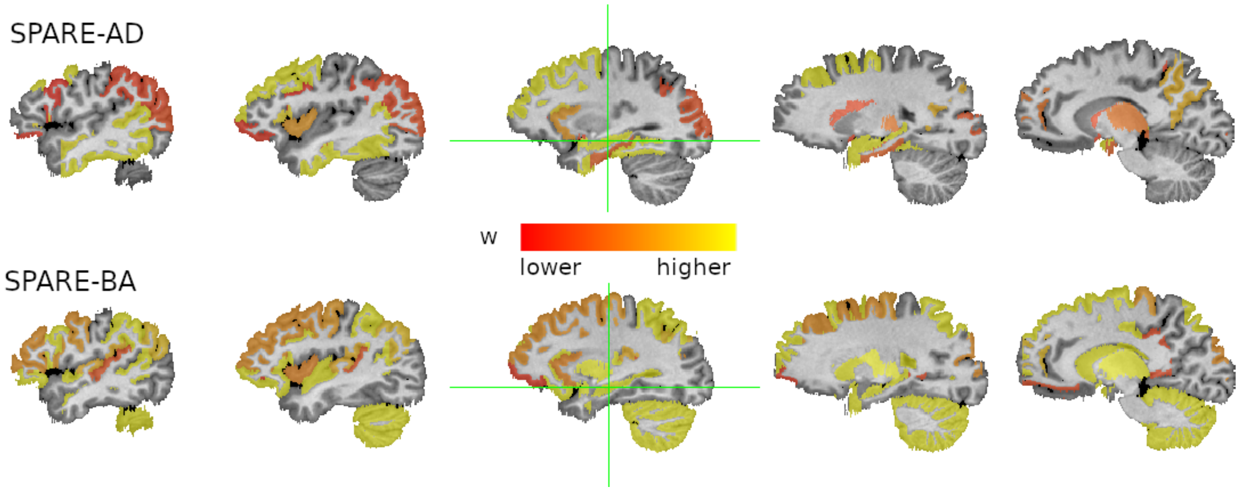

Supplementary Table 1: MRI Acquisition Protocols for Each Site

Study	*Scanner*	*T1 protocol*	*T2-FLAIR protocol*
**ACS**	3T<br>2 scanners<br>Siemens Tim Trio,<br>Biograph mMR	MPRAGE<br>1 × 1 × 1mm<br>Flip angle = 8^0<br>TE = 3.16 ms<br>TR = 2400 ms<br>TI = 1000 ms	Axial_T2-FLAIR<br>.85 × .85× 5.0 mm<br>Flip angle = 150^0<br>TE = 910 ms<br>TR = 9000 ms<br>TI = 2500 ms
**AIBL**	1.5T and 3T<br>4 scanners<br>Siemens Avanto, Skyra,<br>TrioTim, Verio	MPRAGE, sagittal<br>1 × 1 × 1.2mm<br>Flip angle = 9^0<br>TE = 2.13 ms / 2.98&3.05 ms<br>TR = 1900 ms / 2300 ms<br>TI = 900 ms	3D FLAIR<br>.98 × .98 × .9mm<br>Flip angle = 120^0<br>TE = 420 ms<br>TR = 6000 ms<br>TI = 2100 ms
**BLSA**	1.5T<br>3 scanners<br>GE Signa	SPGR<br>.94 × .94 × 1.5 mm<br>Flip angle = 45^0<br>TE = 5 ms<br>TR = 35 ms<br>TI = 0.0 ms	N/A
**BLSA**	1.5T<br>1 scanner<br>Phillips Achieva	SPGR<br>.94 × .94 × 1.5 mm<br>Flip angle = 45^0<br>TE = 5 ms<br>TR = 35 ms<br>TI = 0.0 ms	T2-FLAIR, axial<br>.938 × .938 × 3.0 mm<br>Flip angle = 90^0<br>TE = 140 ms<br>TR = 11000 ms<br>TI = 2725 ms
**BLSA**	3T<br>3 scanners<br>Philips Achieva	MPRAGE, sagittal<br>1 × 1 × 1.2 mm<br>Flip angle = 8^0<br>TE = 3.2 ms<br>TR = 6.5 ms or 6.8 ms<br>TI = 0.0 ms	T2-FLAIR, axial<br>.83 × .83 × 4.4 mm<br>Flip angle = 90^0<br>TE = 68 ms<br>TR = 11000 ms<br>TI = 2800 ms
**BIOCARD**	1.5T<br>1 scanner<br>GE Genesis Signa	SPGR, axial<br>1 x 1 x 2 mm<br>flip angle = 20^0<br>TE = 2 ms<br>TR = 24 ms<br>TI = 0.0ms	FLAIR, axial<br>1 x 1 x 5 mm<br>flip angle = 90^0<br>TR=9002<br>TE=157.5,<br>TI=2200 ms
**BIOCARD**	3T<br>1 scanner<br>Phillips Achieva	MPRAGE<br>1 × 1 × 1.2 mm<br>Flip angle = 8^0<br>TE = 3.1 ms<br>TR = 6.75 ms<br>TI = 0.0 ms	FLAIR<br>1 × 1 × 2 mm<br>Flip angle = 90^0<br>TE = 100 ms<br>TR = 11000 ms<br>TI = 2800 ms
**WRAP**	3T<br>2 scanners<br>GE Discovery,<br>Signa Premiere	SPGR, axial<br>1 × 1 × 1 mm<br>Flip angle = 12^0<br>TE = 3.2 ms<br>TR = 8.2 ms<br>TI = 450 ms	3D FLAIR, sagittal<br>1 × 1 × 2 mm<br>Flip angle = 90^0<br>TE = 123 ms<br>TR = 6000 ms<br>TI = 1868 ms

Supplementary Table 2: Baseline characteristics of participants in APOE analyses with volumetric data, by cohort

	**ACS**	**AIBL**	**BIOCARD**	**BLSA**	**WRAP**	**p-value**
N	299	585	221	189	247	
Age at baseline MRI scan, M (SD)	61.6 (8.0)	72.4 (6.3)	57.3 (9.9)	70.3 (8.5)	61.7 (6.1)	<0.001
Female sex, N (%)	189 (63.2%)	333 (56.9%)	136 (61.5%)	94 (49.7%)	177 (71.7%)	<0.001
Years of education, M (SD)	16.2 (2.4)	12.9 (3.0)	17.3 (2.3)	17.0 (2.1)	16.1 (2.2)	<0.001
MMSE score, M (SD)	29.3 (1.0)	28.6 (1.4)	29.4 (0.9)	28.9 (1.2)	29.3 (0.9)	<0.001
Progressed to MCI/dementia, N (%)	12 (4.0%)	24 (4.1%)	36 (16.3%)	20 (10.6%)	2 (0.8%)	<0.001
Vascular Risk Score, M (SD)	1.2 (1.1)	1.1 (1.0)	0.9 (1.0)	1.4 (1.1)	1.0 (1.0)	<0.001
Vascular Risk Score >=1, N (%)	207 (69.2%)	409 (69.9%)	123 (55.7%)	150 (79.4%)	149 (60.3%)	<0.001
Vascular Risk Score >=2, N (%)	95 (31.8%)	180 (30.8%)	56 (25.3%)	84 (44.4%)	66 (26.7%)	<0.001
Vascular Risk Score >=3, N (%)	37 (12.4%)	57 (9.7%)	22 (10.0%)	33 (17.5%)	15 (6.1%)	0.003
**Genetic factors**						
APOE ε2 carriers, N (%)[a]	33 (11.0%)	78 (13.3%)	26 (11.8%)	24 (12.7%)	23 (9.4%)	0.78
APOE ε4 carriers, N (%)[b]	107 (35.8%)	167 (28.5%)	73 (33.0%)	56 (29.6%)	92 (37.2%)	0.064
APOE ε3/3 carriers, N (%)	159 (53.2%)	340 (58.1%)	122 (55.2%)	109 (57.7%)	132 (53.4%)	0.56
APOE ε3/4 carriers, N (%)	81 (27.1%)	132 (22.6)	54 (24.4%)	46 (24.3%)	72 (29.4%)	0.25
APOE ε4/4 carriers, N (%)	16 (5.4%)	21 (3.6%)	15 (6.8%)	2 (1.1%)	9 (3.6%)	0.037
APOE ε2/4 carriers, N (%)	10 (3.3%)	14 (2.4%)	4 (1.8%)	8 (4.2%)	10 (4.0%)	0.42
**MRI measures**						
SPARE_AD, M (SD)	-1.6 (0.7)	-1.1 (0.8)	-1.4 (0.9)	-1.3 (0.8)	-1.5 (0.7)	<0.001
SPARE_BA, M (SD)	62.8 (9.9)	72.3 (8.8)	61.7 (13.5)	69.2 (10.5)	61.1 (8.8)	<0.001
SPARE_BA residual, M (SD)	0.4 (6.6)	-0.0 (7.1)	-0.3 (7.7)	-1.3 (7.0)	-0.9 (7.1)	0.053
Hippocampal volume, M (SD) in mm^3	3820 (398)	3714 (414)	3792 (412)	3696 (381)	3851 (374)	<0.001
WMH volume (in mm3), M (SD)	1125 (1905)	3695 (6647)	1970 (3737)	4066 (7341)	1786 (2476)	<0.001
Number of MRI measures over time, M (SD) [range]	2.5 (1.3) [1-7]	2.2 (1.6) [1-7]	3.7 (1.6) [1-8]	6.5 (4.4) [1-18]	2.3 (1.2) [1-5]	<0.001
Years between baseline and last MRI, M (SD) [range]	4.5 (3.6) [0-12.3]	2.3 (2.9) [0-10.5]	12.6 (6.5) [0-22.2]	9.3 (6.6) [0-24.4]	3.6 (3.2) [0-8.8]	<0.001

[a] includes ε2/ε2 and ε2/ε3 carriers

[b] includes ε2/ε4, ε3/ε4, and ε4/ε4 carriers

Note: Differences in baseline participant characteristics across cohorts was assessed using a global *F* test for continuous variables or a global χ^2 test for categorical variables, as indicated by the *p*-value column in the table. Global tests, which examine variability across all groups simultaneously, were used to protect against false-positive results.

Some of the differences in cohort characteristics, such as baseline age and *APOE-ε4* genetic status, reflect differences in study design. For example, the proportion of *APOE-ε4* carriers in AIBL is in line with the general population, whereas the three cohorts (ACS, BIOCARD, WRAP) with an overrepresentation of *APOE-ε4* carriers were enriched for a family history of Alzheimer's disease dementia, by design. Additionally, these three cohorts have younger baseline ages because these studies enrolled individuals who were largely middle-aged at baseline, also by design. To help adjust for differences across cohorts, all mixed effect models included separate indicators for each cohort and cohort x time interaction terms.

Supplementary Table 3: Baseline characteristics of participants in AD-PRS analyses with volumetric data, by cohort

	**ACS**	**AIBL**	**BIOCARD**	**BLSA**	**WRAP**	**p-value**
N	199	397	161	106	230	
Age at baseline MRI scan, M (SD)	61.6 (8.1)	73.0 (6.0)	57.8 (9.7)	69.7 (7.8)	61.9 (6.2)	<0.001
Female sex, N (%)	124 (62.3%)	224 (56.4%)	101 (62.7%)	58 (54.7%)	165 (71.7%)	0.002
Years of education, M (SD)	16.2 (2.4)	12.6 (2.9)	17.1 (2.4)	16.8 (2.1)	16.1 (2.2)	<0.001
MMSE score, M (SD)	29.3 (1.0)	28.8 (1.3)	29.4 (1.0)	29.1 (1.0)	29.3 (1.0)	<0.001
Progressed to MCI/dementia, N (%)	10 (5.0%)	20 (5.0%)	27 (16.8%)	12 (11.3%)	2 (0.9%)	<0.001
Vascular Risk Score, M (SD)	1.1 (1.0)	1.1 (1.0)	1.0 (1.1)	1.3 (1.0)	1.0 (1.0)	0.028
Vascular Risk Score >=1, N (%)	138 (69.4%)	277 (69.8%)	89 (55.3%)	83 (78.3%)	140 (56.7%)	<0.001
Vascular Risk Score >=2, N (%)	56 (28.1%)	118 (29.7%)	49 (30.4%)	43 (40.6%)	60 (24.3%)	0.10
Vascular Risk Score >=3, N (%)	19 (9.6%)	32 (8.1%)	20 (12.4%)	12 (11.3%)	15 (6.1%)	0.26
**Genetic factors**						
APOE ε2 carriers, N (%)[a]	21 (10.6%)	59 (14.9%)	15 (9.3%)	9 (8.5%)	21 (9.2%)	0.46
APOE ε4 carriers, N (%)[b]	75 (37.7%)	96 (24.2%)	53 (32.9%)	28 (26.4%)	87 (37.8%)	<0.001
APOE ε3/3 carriers, N (%)	103 (51.8%)	242 (61.0%)	93 (57.8%)	69 (65.1%)	122 (53.0%)	0.06
APOE ε3/4 carriers, N (%)	59 (29.6%)	74 (18.6%)	39 (24.2%)	23 (21.7%)	68 (29.7%)	0.008
APOE ε4/4 carriers, N (%)	10 (5.0%)	15 (3.8%)	11 (6.8%)	1 (0.9%)	9 (3.9%)	0.19
APOE ε2/4 carriers, N (%)	6 (3.0%)	7 (1.8%)	3 (1.9%)	4 (3.8%)	10 (4.3%)	0.33
**MRI Measures**						
SPARE_AD, M (SD)	-1.6 (0.7)	-1.1 (0.8)	-1.4 (0.8)	-1.4 (0.8)	-1.5 (0.7)	<0.001
SPARE_BA, M (SD)	62.7 (9.9)	72.7 (9.0)	63.3 (12.9)	68.8 (9.1)	61.4 (8.9)	<0.001
SPARE_BA residual, M (SD)	0.4 (6.5)	-0.3 (7.2)	0.0 (7.8)	-1.2 (6.2)	-0.8 (7.2)	0.27
Hippocampal volume, M (SD) in mm^3	3840 (409)	3691 (393)	3787 (412)	3717 (410)	3848 (373)	<0.001
WMH volume (in mm3), M (SD)	10076 (1850)	4308 (7979)	2027 (3317)	3366 (5296)	1863 (2535)	<0.001
Number of MRI measures over time, M (SD) [range]	2.5 (1.3) [1-7]	2.5 (1.6) [1-7]	3.8 (1.6) [1-8]	7.3 (4.7) [1-18]	2.3 (1.2) [1-5]	<0.001
Years between baseline and last MRI, M (SD) [range]	4.5 (3.6) [-0.9-12.3]	3.0 (3.0) [-0.8-10.4]	13.0 (6.4) [-0.8-22.1]	10.5 (6.9) [-0.02-24.4]	3.5 (3.3) [-0.9-8.6]	<0.001

[a] includes ε2/ε2 and ε2/ε3 carriers

[b] includes ε2/ε4, ε3/ε4, and ε4/ε4 carriers

Note: Differences in baseline participant characteristics across cohorts was assessed using a global *F* test for continuous variables or a global χ^2 test for categorical variables, as indicated by the *p*-value column in the table. Global tests, which examine variability across all groups simultaneously, were used to protect against false-positive results.

Supplementary Table 4: Mixed-effects model results from fully-adjusted model of *APOE* genetic status in relationship to MRI measures.

	**SPARE-AD (AD-related atrophy)**		**SPARE-BA-resid (age-related atrophy)**		**Hippocampus volume**		**WMH volume**	
Predictor	Estimate (SE)	*p*-value	Estimate (SE)	*p*-value	Estimate (SE)	*p-value*	Estimate (SE)	*p-value*
Time	-0.139 (0.025)	<0.0001	-0.031 (0.022)	0.16	0.078 (0.018)	<0.0001	-0.089 (0.023)	<0.0001
$Time^2$	0.003 (0.0003)	<0.0001	-0.002 (0.0003)	<0.0001	-0.000 (0.0002)	0.11	--	--
age	0.037 (0.003)	<0.0001	-0.131 (0.003)	<0.0001	-0.035 (0.003)	<0.0001	0.050 (0.003)	<0.0001
age x time	0.003 (0.0004)	<0.0001	0.001 (0.0003)	<0.0001	-0.002 (0.0003)	<0.0001	0.002 (0.0003)	<0.0001
Sex (female)	0.005 (0.049)	0.91	0.022 (0.053)	0.68	-0.057 (0.048)	0.24	-0.018 (0.050)	0.71
sex (F) x time	-0.015 (0.006)	0.009	-0.027 (0.005)	<0.0001	0.007 (0.004)	0.09	0.007 (0.005)	0.22
education	0.015 (0.025)	0.53	0.034 (0.026)	0.20	0.011 (0.024)	0.66	-0.000 (0.025)	0.99
education x time	-0.005 (0.003)	0.08	-0.003 (0.002)	0.18	-0.000 (0.002)	0.87	-0.002 (0.003)	0.53
VRS	0.032 (0.014)	0.026	0.020 (0.015)	0.19	0.001 (0.011)	0.96	-0.007 (0.011)	0.55
VRS x time	-0.003 (0.002)	0.05	0.000 (0.0004)	0.81	0.001 (0.001)	0.48	-0.002 (0.002)	0.48
Progressed	0.061 (0.105)	0.56	0.012 (0.008)	0.13	-0.128 (0.102)	0.21	0.260 (0.104)	0.012
Progressed x time	0.088 (0.009)	<0.0001	0.012 (0.008)	0.13	-0.049 (0.007)	<0.0001	0.024 (0.010)	0.012
APOE-ε2	-0.037 (0.066)	0.58	0.021 (0.071)	0.77	0.010 (0.065)	0.88	0.010 (0.067)	0.88
APOE-ε2 x time	0.001 (0.007)	0.85	0.000 (0.006)	1.00	0.005 (0.005)	0.34	0.017 (0.007)	0.011
APOE-ε4	0.072 (0.051)	0.16	-0.058 (0.055)	0.29	-0.007 (0.050)	0.90	-0.051 (0.052)	0.33
APOE-ε4 x time	0.015 (0.006)	0.011	0.010 (0.005)	0.04	-0.013 (0.004)	0.001	0.007 (0.005)	0.19

Note. VRS = Vascular Risk Score.
All linear mixed-effects models included separate indicators for each cohort and cohort x time interaction terms to help adjust for cohort differences.

Supplementary Table 5: Mixed-effects model results of dichotomous AD polygenic risk score and *APOE* genetic status in relationship to MRI measures.

	SPARE-AD (AD-related atrophy)		SPARE-BA-resid (age-related atrophy)		Hippocampus volume		WMH volume	
	Estimate (SE)	*p-value*	*Estimate (SE)*	*p-value*	*Estimate (SE)*	*p-value*	*Estimate (SE)*	*p-value*
AD-PRS	-0.107 (0.065)	0.102	-0.036 (0.071)	0.62	-0.012 (0.067)	0.86	-0.198 (0.069)	0.004
AD-PRS x time	0.015 (0.008)	0.054	0.005 (0.006)	0.43	-0.015 (0.005)	0.007	0.006 (0.007)	0.34
APOE-ε2	0.060 (0.079)	0.45	0.061 (0.086)	0.48	-0.090 (0.081)	0.27	0.074 (0.084)	0.38
APOE-ε2 x time	-0.000 (0.010)	0.99	-0.004 (0.008)	0.65	0.006 (0.007)	0.35	0.008 (0.008)	0.33
APOE-ε4	0.132 (0.061)	0.030	-0.047 (0.066)	0.48	-0.009 (0.062)	0.88	0.029 (0.064)	0.66
APOE-ε4 x time	0.021 (0.007)	0.004	0.010 (0.006)	0.10	-0.021 (0.005)	<0.0001	0.012 (0.006)	0.049

Note: All models were adjusted by baseline age, sex, years of education, indicators for each cohort, and included interactions of each predictor with time (e.g., terms for all genetic predictors x time and covariates x time). AD-PRS scores were dichotomized into high (upper 25%) vs. low (lower 75%).

Supplementary Table 6: Mixed-effects model results for interactions between *APOE* genetic status and AD-PRS scores in relationship to level and change in MRI measures.

	**SPARE-AD (AD-related atrophy) n=1,063**		**SPARE-BA-resid (age-related atrophy) n=1,063**		**Hippocampus volume n=1,063**		**WMH volume n=948**	
	Estimate (SE)	*p-value*	*Estimate (SE)*	*p-value*	*Estimate (SE)*	*p-value*	*Estimate (SE)*	*p-value*
AD-PRS x APOE-ε2	0.116 (0.097)	0.23	0.040 (0.105)	0.70	-0.040 (0.099)	0.69	-0.057 (0.102)	0.58
AD-PRS x APOE-ε2 x time	-0.000 (0.012)	0.98	-0.000 (0.010)	0.96	-0.005 (0.009)	0.57	0.009 (0.011)	0.41
AD-PRS x APOE-ε4	0.018 (0.064)	0.29	-0.055 (0.070)	0.43	-0.012 (0.066)	0.85	-0.041 (0.069)	0.56
AD-PRS x APOE-ε4 x time	-0.002 (0.008)	0.78	0.004 (0.006)	0.47	-0.003 (0.005)	0.58	0.014 (0.007)	0.038

Note: These models excluded APOE-ε2/ε4 carriers to simply interpretation, though results very similar when these participants were included (data not shown). All models included the AD-PRS score, indicators for *APOE-*ε2 and *APOE-ε4,* their interactions with time, and were adjusted for baseline age, sex, years of education, cohort, and their interactions with time.

Supplementary Text 1: Additional information on SPARE-BA and SPARE-AD

SPARE stands for "Spatial Pattern of Abnormalities for Recognition of…". SPARE-AD scores represent the degree to which an individual's structural brain pattern includes features of AD-like brain atrophy. SPARE-AD scores have been validated previously (Da et al., 2014; Habes et al., 2016). The model was constructed to maximally differentiate between MRI scans from amyloid-positive AD-dementia participants (n=221) vs. age-matched amyloid negative cognitively unimpaired (CU) participants (n=256) from the ADNI using a support vector machine (SVM). More positive SPARE-AD implies a more AD-like brain structure, while more negative values reflect more normal brain structure. For SPARE-AD calculation, an SVM classifier was trained with a linear kernel to predict the diagnosis status as either CU or AD. For predicted classes of the training set, cross-validation was performed using the predictions from each holdout fold to avoid over-fitting. The SPARE-AD classifier attained an ROC area-under-the-curve score of 0.948 and Accuracy of 0.893 based on the cross-validated predictions. As published previously, individuals with high SPARE-AD scores tend to have lower gray matter volumes most pronounced in the hippocampus, amygdala, entorhinal cortex, and inferior temporal cortex (see Supplementary Figure 1). Cognitively normal individuals with higher SPARE-AD scores also have lower executive function and episodic memory scores, particularly after age 65 (Habes et al., 2020).

SPARE-BA scores have also been previously validated (Eavani et al., 2018; Habes et al., 2016). Higher SPARE-BA values indicate greater age-related atrophy compared to normative trends of age-related changes in brain structure. To calculate SPARE-BA scores, a multivariate pattern regression model based on support vector regression was used to predict individualized brain age for each participant. The model was trained with the T1-MR scans using regional volumetric measures for structures. The training set included only cognitively normal subjects. Prior work has shown that advanced brain aging (defined as SPARE-BA scores 5+ years older than chronological age) vs. resilient brain aging (defined as SPARE-BA scores 5+ years younger than chronological age) is associated with widespread lower gray matter volumes, most pronounced in the frontal operculum, superior temporal, insular, and frontal and inferior parietal cortex, in addition to enlargement of the ventricles. Additionally, cognitively normal individuals with more advanced brain aging have lower scores on tests of executive function but not on tests of episodic memory (Habes et al., 2020).

The scripts necessary for applying the SPARE model, along with the pre-trained SPARE models used to calculate SPARE scores in the PAC dataset, are publicly available through an open-source software package hosted on GitHub (https://github.com/CBICA/spare_score). Additionally, the SPARE package is offered as both a software container and a cloud application accessible at https://neuroimagingchart.com/."

Of note, the SPARE-BA model was trained on cross-sectional data, as is typical of brain age models, and the derived weights were then applied to longitudinal data. Future studies should examine whether it is possible to construct a brain age model of the rate of brain change using longitudinal data. Such a "second-order" changing brain age model may provide additional insights regarding age-related brain changes, but will require large samples from multiple studies with sufficient longitudinal data.

References

Da X, Toledo JB, Zee J, et al. Integration and relative value of biomarkers for prediction of MCI to AD progression: Spatial patterns of brain atrophy, cognitive scores, APOE genotype and CSF biomarkers. NeuroImage Clin. 2014;4(0):164-173.

Eavani H, Habes M, Satterthwaite TD, et al. Heterogeneity of structural and functional imaging patterns of advanced brain aging revealed via machine learning methods. Neurobiol Aging. 2018;71:41-50.

Habes M, Janowitz D, Erus G, et al. Advanced Brain Aging: relationship with epidemiologic and genetic risk factors, and overlap with Alzheimer disease atrophy patterns. Transl Psychiatry. 2016;6:e775.

Supplementary Text 2: Formal model comparison of APOE-/AD-PRS x time interaction terms for SPARE AD and SPARE-BA

Although not a primary aim of the current study, we compared the effect of the (APOE-ε4 x time) term for SPARE-AD vs. SPARE-BA. To do so, we modeled both outcomes simultaneously in a combined linear mixed effects model with an indicator variable for the dependent variable (SPARE-BA vs. SPARE-AD). This model included interaction terms of this indicator variable with all predictors (e.g., age, sex, time, education, APOE-ε4, APOE-ε2, AD-PRS, and their interactions with time) to allow for different effects for the two different outcomes. The model included a random intercept and random slopes over time for participants, as well as a random intercept by outcomes and a random slope over time for the outcomes, and independent residual variance estimates by each outcome.

The results of the model showed that the estimated effect of (APOE-ε4 x time) is 0.007 units higher on SPARE-AD compared to SPARE-BA (p=0.329), and the effect of (AD-PRS x time) on SPARE-AD is 0.046 unit lower than for (APOE-ε4 x time), p=0.216. Although the results from this analysis suggest that the strength of the association between APOE-ε4 genetic status with the rate of change in SPARE-AD is not significantly different from the strength of the association of APOE-ε4 with rate of change in SPARE-BA, it is important to keep in mind that results may differ depending on the types and number of covariates included. Additionally, the complexity of the model calls for careful interpretation.
